# Supplementary material for: Human Pathogenic Candida Species Respond Distinctively to Lactic Acid Stress
Source: J Fungi (Basel). 2020 Dec 8;6(4):348. doi: 10.3390/jof6040348 (PMC7762603; doi:10.3390/jof6040348)
Supplement: Supplementary file 1 [file jof-06-00348-s001.zip › Supplements/supplementary_fig_with_caption.pdf]

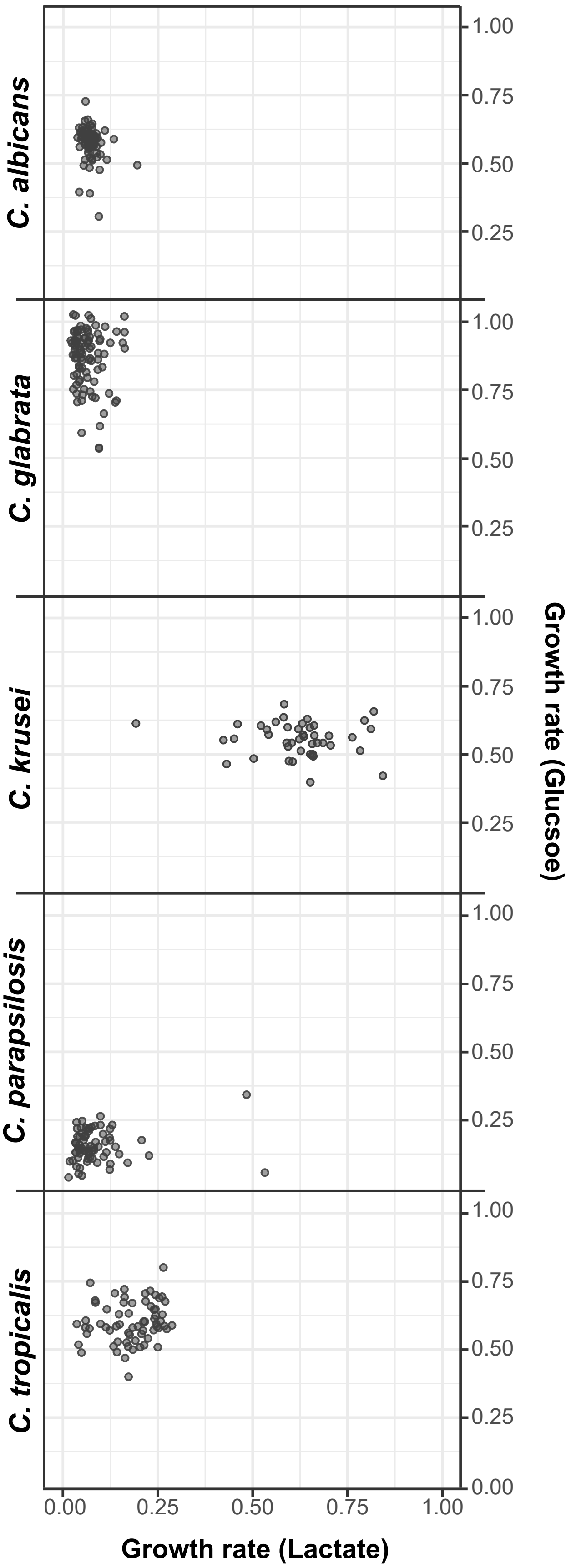

Figure S1. Growth behavior of diverse *Candida* isolates on 2% Lactate or 2% Glucose as sole carbon source at pH 4; Every dot represents one isolate; Experiment was done in at least triplicates; Mean values of maximal growth rates are displayed.
