# Supplementary figures and images for: Human Pathogenic Candida Species Respond Distinctively to Lactic Acid Stress
Source: J Fungi (Basel). 2020 Dec 8;6(4):348. doi: 10.3390/jof6040348 (PMC7762603; doi:10.3390/jof6040348)

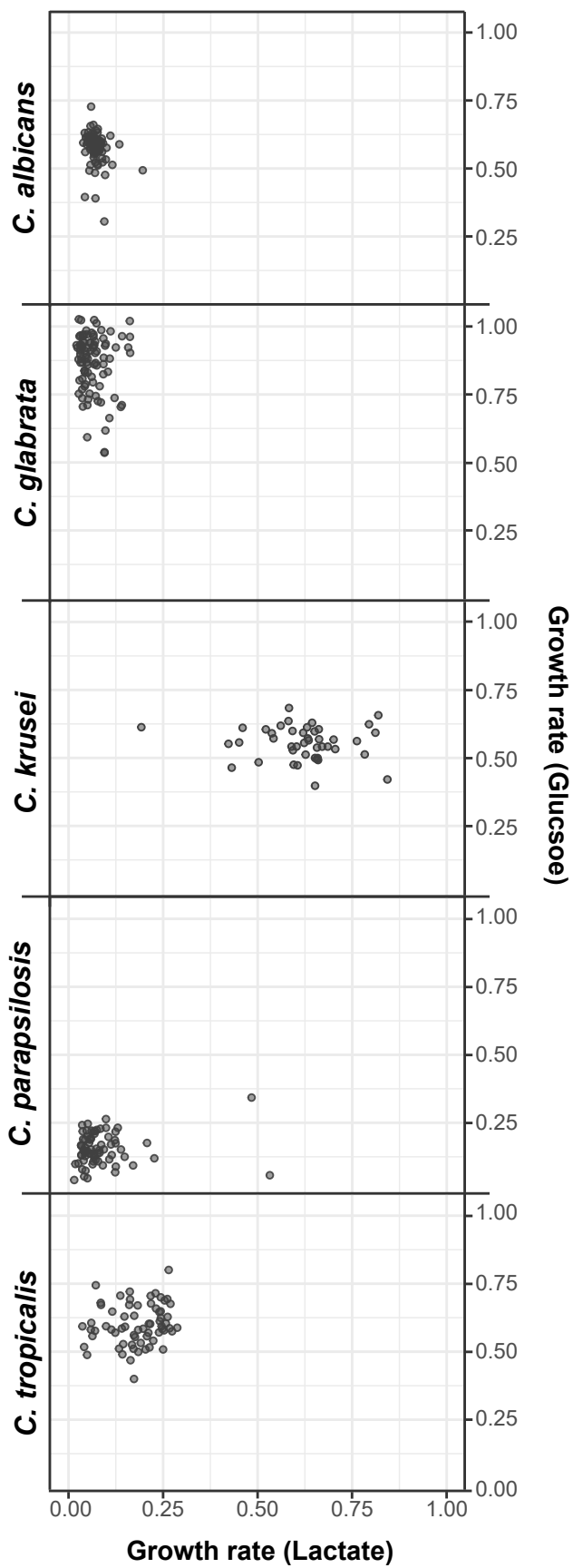

Supplement: Supplementary file 1 [file jof-06-00348-s001.zip › Supplements/Supplementary_Fig_S1.pdf]
